# Genomic imbalances in patients with a clinical presentation in the spectrum of Cornelia de Lange syndrome

**DOI:** 10.1186/1471-2350-14-41

**Published:** 2013-04-03

**Authors:** Cristina Gervasini, Chiara Picinelli, Jacopo Azzollini, Daniela Rusconi, Maura Masciadri, Anna Cereda, Cinzia Marzocchi, Giuseppe Zampino, Angelo Selicorni, Romano Tenconi, Silvia Russo, Lidia Larizza, Palma Finelli

**Affiliations:** 1 Medical Genetics, Department of Health Sciences, Università degli Studi di Milano, Milan, Italy; 2 Laboratory of Medical Cytogenetics and Molecular Genetics, Istituto Auxologico Italiano, Milan, Italy; 3 Pediatric Department, Università Milano Bicocca, Fondazione MBBM, S. Gerardo Hospital, Monza, Italy; 4 Genetica Clinica Epidemiologica, Dipartimento Pediatria, Padua, Italy; 5 Department of Pediatrics, Catholic University, Rome, Italy; 6 Deptartment of Medical Biotechnology and Translational Medicine, Università degli Studi di Milano, Milan, Italy; 7 Department of Medical Biotechnology and Translational Medicine, C/O Laboratory of Medical Cytogenetics and Molecular Genetics, Centro d Ricerche e Tecnologie Biomediche, IRCCS-Istituto Auxologico Italiano, via Zucchi 18, Cusano Milanino, MI 20095, Italy

## Abstract

**Background:**

Cornelia de Lange syndrome (CdLS) is a rare autosomal-dominant disorder characterised by facial dysmorphism, growth and psychomotor developmental delay and skeletal defects. To date, causative mutations in the *NIPBL* (cohesin regulator) and *SMC1A* (cohesin structural subunit) genes account for > 50% and 6% of cases, respectively.

**Methods:**

We recruited 50 patients with a CdLS clinical diagnosis or with features that overlap with CdLS, who were negative for mutations at *NIPBL* and *SMC1A* at molecular screening. Chromosomal rearrangements accounting for the clinical diagnosis were screened for using array Comparative Genomic Hybridisation (aCGH).

**Results:**

Four patients were shown to carry imbalances considered to be candidates for having pathogenic roles in their clinical phenotypes: patient 1 had a 4.2 Mb *de novo* deletion at chromosome 20q11.2-q12; patient 2 had a 4.8 Mb deletion at chromosome 1p36.23-36.22; patient 3 carried an unbalanced translocation, t(7;17), with a 14 Mb duplication of chromosome 17q24.2-25.3 and a 769 Kb deletion at chromosome 7p22.3; patient 4 had an 880 Kb duplication of chromosome 19p13.3, for which his mother, who had a mild phenotype, was also shown to be a mosaic.

**Conclusions:**

Notwithstanding the variability in size and gene content of the rearrangements comprising the four different imbalances, they all map to regions containing genes encoding factors involved in cell cycle progression or genome stability. These functional similarities, also exhibited by the known CdLS genes, may explain the phenotypic overlap between the patients included in this study and CdLS. Our findings point to the complexity of the clinical diagnosis of CdLS and confirm the existence of phenocopies, caused by imbalances affecting multiple genomic regions, comprising 8% of patients included in this study, who did not have mutations at *NIPBL* and *SMC1A*. Our results suggests that analysis by aCGH should be recommended for CdLS spectrum cases with an unexplained clinical phenotype and included in the flow chart for diagnosis of cases with a clinical evaluation in the CdLS spectrum.

## Background

Cornelia de Lange syndrome (CdLS) is a rare, genetically heterogeneous (OMIM #122470, #300590 and #610759), multiple congenital anomaly/intellectual disability disease [1,2], characterised by distinctive facial dysmorphism, pre- and post-natal growth deficiency, psychomotor delay, intellectual disability and malformations of the upper limbs (ranging from small hands to complete limb reduction). CdLS also often involves specific medical complications such as gastroesophageal reflux, hypoacusia and seizures. Its clinical presentation ranges from mild/borderline to severe [3], and this variability has led to the definition of a list of consensus diagnostic criteria integrated into a global scoring system of phenotype severity [4], which are an accepted standard [5-7].

Known CdLS-associated genes encode structural and regulatory proteins of the cohesin pathway, which is involved in chromosome segregation, DNA repair, gene expression and chromosome conformation [8]. The first major gene to be identified was *NIPBL*, which is located at chromosome 5p13.2, encodes a member of the adherin family [9,10], and mutations in this gene are responsible for > 50% of CdLS patients. All types of *NIPBL* point mutations have been described, although truncating mutations are generally associated with a more severe phenotype than missense and regulatory mutations [5,9-24]. Microdeletions involving one or more exons of the *NIPBL* genomic region, and large rearrangements extending to the *NIPBL* flanking regions, and correlating with severe syndromic presentation, have also been reported [25-27].

Locus heterogeneity in CdLS has been demonstrated by the X-linked form caused by mutation of the *SMC1A* gene, which encodes a subunit of the cohesin complex [28]. *SMC1A* alterations contribute up to 6% of all CdLS cases and include only missense mutations or in-frame deletions that preserve the protein reading frame [14,24,28-31]. So far, only one patient has been found to have a mutation in the *SMC3* gene, which encodes the other SMC cohesin component, and its epidemiological impact has not yet been defined [29]. Very recently, mutations in the *HDAC8* gene, a vertebrate SMC3 deacetylase, have been identified in CdLS probands [32], and mutations in *RAD21* gene have been found in six patients with CdLS features [33]. The remaining CdLS cases may be due to as yet undetected mutations in the known genes or by other causative anomalies.

The genomic technology of array Comparative Genomic Hybridisation (aCGH), which monitors losses or gains in chromosome regions that may harbour novel candidate genes, is not yet a standard test for investigation of *NIPBL*- and *SMC1A*-mutation-negative CdLS patients [11,18,34,35], but results obtained with the technique to date are consistent with those of > 30 conventional cytogenetic and FISH-targeted studies that have shown chromosomal abnormalities associated with the CdLS phenotype involving almost all of the chromosomes (reviewed [36]). One study has used aCGH to study probands with CdLS-like features, who had been previously screened for mutations in the two major causative genes; however, this was performed in a relatively small patient cohort [35].

This aCGH study of 50 probands including patients fulfilling CdLS diagnostic criteria and those not completely fulfil the criteria [4], and negative for mutations at the *NIPBL* and *SMC1A* loci, led to the detection of four carriers of large genomic imbalances that are candidates to explain the clinical phenotype and represent a fraction (8%) of patients with features overlapping those of CdLS. We herein describe how the analysis of the gene content of these imbalances, affecting different genomic regions, links the altered dosage of specific gene classes, shared by all rearrangements, to a common CdLS-like phenotype.

## Methods

### Patients

CdLS is characterized by a wide phenotypic spectrum; despite some features are quite typical the patients present with a highly variable phenotype ranging from severe to very mild. Out of the fifty probands (26 males and 24 females) investigated in this study diagnosis by our clinical geneticists (AS, RT, GZ) was CdLS for those (60%) fulfilling the international CdLS diagnosis criteria [4] or CdlS-like for the remaining (40%,) not fully satisfying the CdLS criteria. According to the CdLS scoring system [4] the overall phenotype of the patients was severe (~10%) or moderate-mild (~90% with slight prevalence of moderate phenotype). All patients were found negative for *NIPBL* and *SMC1A* mutations by DHPLC, direct sequencing and MLPA analyses.

Written informed consent to the research study, which was approved by the Ethical Clinical Research Committee of Istituto Auxologico Italiano, and to the publication of the face photo(s) was obtained from one of the parents.

### Array-CGH analysis

The probands were investigated by means of aCGH. Genome scans were performed using the Human Genome CGH Microarray Kit 244 K (Agilent Technologies, Palo Alto, CA), which consists of ~236,000 60-mer oligonucleotide probes covering the entire genome at an average spatial resolution of ~30 kb. The samples were labelled and hybridised following the protocols provided by Agilent, and the arrays were analysed using the Agilent Scanner Control (v 7.0) and Feature Extraction software (v 9.5.1). Graphical overviews were obtained using CGH Analytic software (v4.0.81). Aberration calls were identified using the ADM-2 algorithm.

An *in silico* analysis of the unbalanced regions indicated by aCGH was performed using the March, 2006, release of the UCSC Genome Browser (http://genome.ucsc.edu/) and the Database of Genomic Variants (http://projects.tcag.ca/variation).

### FISH

Chromosome preparations were obtained by standard cytogenetic techniques using peripheral blood lymphocytes cultured by 72 h. BAC probes were selected on the basis of their physical location (http://www.genome.ucsc.edu/ release March, 2006), and provided by Invitrogen Ltd., UK. Their physical positions were verified on control metaphase chromosomes derived from peripheral blood lymphocytes. FISH experiments were performed using standard procedures [37].

## Results

The 50 probands negative for *NIPBL* and *SMC1A* mutations were considered an ideal cohort to scan for the presence of genomic gains/losses by aCGH, in the search for novel genes responsible for phenotypes with features that overlap CdLS.

We identified four probands with large or *de novo* copy number variants (CNVs) (Table 1), whose clinical data at birth and at age of evaluation are summarised in Table 2 (see also Additional file 1: Table S1 for auxological parameters). The four probands display a high degree of phenotypic heterogeneity, but all share the minimal diagnostic CdLS clinical features. Three of them (Probands 1, 2 and 3) fulfil the diagnostic CdLS criteria, whereas the fourth (Proband 4) does not fulfil completely the criteria having synophrys and only two (and not three) other facies criteria (Additional file 2: Table S2).

**Table 1:** **Chromosomal position and boundaries of large rearrangements identified by aCGH in four CdLS probands negative for mutations in *NIPBL *and *SMC1A***

***Pt***	***Rearrangement***	***Size***	Boundaries (bp)*	***Origin***
1	del(20)(q11.2q12)	4.2 Mb (min 4.259-max 4.313)	chr20:33228486-37488426	de novo**
2	del(1)(p36.23p36.22)	4.8 Mb (min 4.889-max 4.914)	chr1:7161146-12049775	de novo**
3	der(7)t(7;17)(p22.3;q24.2)	769 Kb del(7)(p22.3) (min 769-max 916)	chr7:140213-909190	t(7;17)(p22.3;q24.2)pat
		14 Mb dup(17)(q24.2q25.3) (min 14.744-max 14.763)	chr17:63665720-78409550	
4	dup(19)(p13.3)	880 Kb (min 880–891)	chr19:662118-1541750	mother: mos dup(19)(p13.3)***

*UCSC Genome Browser assembly Mar.2006 (NCBI36/hg18).

**Paternity confirmed by inheritance of benign CNVs.

***Mosaic condition.

**Table 2:** Spectrum of clinical features in CdLS probands carrying imbalances compared to those of classic CdLS probands

**Clinical features**	**CdLS**	**1**	**2**	3*	4**	**4**′**
		**del(20)(q11.2q12)**	**del(1)(p36.23p36.22)**	**der(7)t(7;17)(p22.3;q24.2)**	**dup(19)(p13.3)**	**mos dup(19)(p13.3)**
**Pre-/post-natal growth retardation**	IUGR; PNGR	IUGR; PNGR	IUGR; PNGR	PNGR; swallowing difficulties	IUGR	NA
**Neurological involvement**	Psychomotor/cognitive impairment; hypertonicity/ hypotonia; seizures (25%)	Psychomotor retardation; hypertonicity	Severe psychomotor and intellectual disability.; hyperactivity	Psychomotor and intellectual disability	Hyperactivity; mild intellectual disability; emotional problems	Dyslexia; bulimia
**Craniofacial appearance**	microbrachycephaly	plagiocephaly	Microcephaly; temporal narrowing	microbrachycephaly		
Face	Long and prominent philtrum; micrognathia (80%)	Normal	Long philtrum; micrognathia	Long face; long and prominent philtrum	Long philtrum	Long hypoplastic philtrum
Eyes Eyelashes Eyebrows	Myopia; long curly eyelashes; synophrys; arched eyebrows	Myopic astigmatism; long eyelashes; synophrys	Long eyelashes; synophrys	Long eyelashes; synophrys; large eyebrows	Synophrys	Synophrys
Nose	Depressed/broad nasal bridge; upturned nasal tip; anteverted nares	Columella below alae nasi	Large nasal tip; anteverted nares	Depressed nasal bridge; large columella	NA	NA
Mouth	Thin upper lip; downturned corners of the mouth; high and arched palate; cleft lip/palate	Thin upper lip; downturned corners of the mouth; arched palate	Large mouth; thin upper lip	Thin upper lip; high palate; downturned corners of the mouth	Thin upper lip	Thin upper lip
Ears	Low-set posteriorly rotated and/or hirsute ears; thickened helices	Bilateral hypoplastic helix	NA	Low-set ears	Ear lobe creases	Ear lobe creases
Hair	Hirsutism (>80%); low posterior hairline	Frontotemporal hypertrichosis and truncal hirsutism	Slight hirsutism	Hirsutism	NA	NA
**Skeleton**	Ranging from severe reduction defects to milder defects such as micromelia, proximally placed thumbs fifth finger clinodactyly, limited elbow extension, syndactyly of the toes, and occasional orthopedic complications (scoliosis)	Normal	Proximally placed thumb; small hands; slight toenail dysplasia	Post-axial polydactyly of left hand and foot#	Clynodactyly of 5th finger	NA
**Cardiovascular**	Cardiac defects (ASD/VSD, …)	Secundum small atrial septal defect	Mitral valve prolapse	Normal	Normal	NA
**Gastrointestinal**	Gastroesophageal reflux (30-80%); congenital diaphragmatic hernia (1%)	Feeding problems in the first year of life	Gastroesophageal reflux	Feeding problems in the first years of life	Normal	NA
**Breast**	Small nipples	Normal	NA	Normal	Polythelia	NA
**Other**		Thenar and hypothenar hypoplasia; bilateral inguinal hernia; hyperactivity	NA	Cryptorchidism	Scoliosis; cryptorchidism	Monolateral hypoplastic kidney
**Age at evaluation**		9 years 10 months	12 years	12 years 6 months	10 years	NA

ASD/VSD: atrial septal defect/ventricular septal defect.

IUGR: intra-uterine growth retardation.

NA not assessed.

OFC Occipitofrontal Circumference.

PNGR: post-natal growth retardation.

# not inherited.

* Balanced translocation inherited from healthy father.

** Familial case (4′: mother of proband 4).

### Proband 1

The first proband was a 9-year-old girl who showed both intra-uterine (IUGR) and post-natal growth retardation (PNGR) (Table 2 and Additional file 1: Table S1).

Post-natal growth was poor and psychomotor development retarded due to feeding problems (sucking and swallowing difficulties), and surgical correction of a bilateral inguinal hernia was performed at 3 months of age. At the age of 3.5 months, she developed behavioural disorders including hyperactivity, frequent outbursts/temper tantrums and self-injurious behaviour, with self-hitting and self-biting.

**Figure 1:**
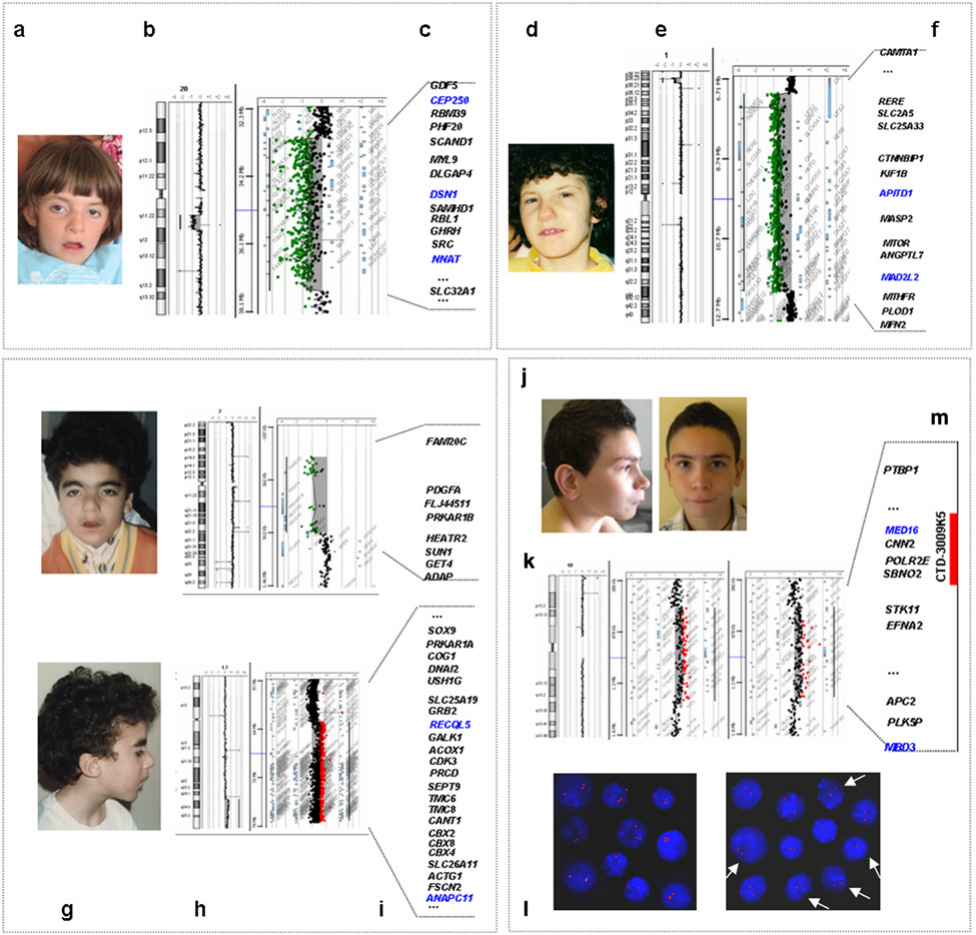
**Facial appearance, genomic imbalances and genes with altered copy number in probands. (a, d, g, j)** The facial appearance of patients 1 (age 6 years), 2 (age 15 years), 3 (age 22 years) and 4 (age 9 years). **(b, e, h, k**) aCGH profiles. (Left) Ideogram of the chromosome(s) involved in the imbalances with the log2 probe ratio plotted as a function of chromosomal position; **h)** profile of patient 4 (left) and his mother (right). **(c, f, i, m)** The gene content of each genomic imbalance. Magnification of the deleted/duplicated region indicating the distal and proximal breakpoint positions (horizontal dotted lines) and a selection of gene content. Blue colour indicates genes cited in the main text. The red bar in **m)** corresponds to the CTD-3009K5 BAC clone used in the FISH analysis (not to scale). **l)** FISH analysis using the CTD-3009K5 BAC clone mapping to chromosome 19p13.3 shows a duplicated signal in all cells of proband 4 (left) and in approximately 76% of the nuclei in a maternal sample, confirming the presence of the rearrangement in a mosaic state (right). White arrows indicate nuclei with a duplicated signal.

When she was 6 years old, a clinical examination revealed generalised hirsutism and dysmorphic features, such as synophrys, long and downward-slanting palpebral fissures, epicanthic folds, long and curved eyelashes, malar hypoplasia, hypoplastic nasal bone, the columella below the *alae nasi*, a thin upper lip and downturned corners of the mouth and a high-arched palate (Figure 1a). She also had slight limb involvement with bilateral thenar and hypothenar hypoplasia. At the age of 9 years and 10 months, she had developed brachycephaly and her dysmorphic facial features were unchanged. Heart sonography revealed a secundum small atrial septal defect. She no longer presented gastrointestinal problems except for mild rectal bleeding due to multiple juvenile polyps (> 15) throughout the colon.

The aCGH analysis showed a large, 4.2 Mb, *de novo* deletion of chromosome 20q11.2-q12 (Table 1, Figure 1b) that was not found in her healthy parents (not shown). More than 50 genes are localised to the deleted region (Figure 1c).

### Proband 2

Proband 2 was a 22-year-old woman who had been affected by both IUGR and PNGR (Table 2 and Additional file 1: Table S1). Clinical evaluation revealed slight hirsutism and dysmorphic features, including microcephaly with temporal narrowing, synophrys, long eyelashes, a large nasal tip, anteverted nares, a long philtrum, a large mouth, a thin upper lip and micrognathia (Figure 1d). Limb involvement was mild, marked by small hands, a proximally placed thumb and slight toenail dysplasia. The patient suffered from gastroesophageal reflux, and mitral valve prolapse.

Neurological assessment showed severe psychomotor and intellectual disability, with some behavioural disorders such as hyperactivity.

The aCGH analysis revealed the presence of a ~4.8 Mb interstitial deletion of chromosome 1p36.23-36.22 1 (Table 1, Figure 1e). Her healthy parents have a normal molecular karyotype (not shown), indicating the *de novo* origin of the rearrangement. The large deleted region includes > 50 genes and does not overlap with the 1p36 syndrome regions (Figure 1f).

### Proband 3

In the first years of life, proband 3, a 22-year-old man, suffered from cryptorchidism and feeding problems with swallowing difficulties, as well as post-natal growth delay and cognitive impairment, with both psychomotor and intellectual disability (Table 2 and Additional file 1: Table S1).

His current phenotype is characterised by hirsutism and facial features consisting of microbrachycephaly, a long face, low-set ears, synophrys, thick eyebrows, long eyelashes, a depressed nasal bridge, a large columella, a long and prominent philtrum, a thin upper lip, a high palate and downturned corners of the mouth (Figure 1g). Limb involvement includes post-axial polydactily of the left hand and foot.

The proband is a carrier of an unbalanced 46,XY translocation der(7)t(7;17)(p22.3;q24.2), inherited from his father, who carries a t(7;17)(p22.3;q24.2) balanced translocation. The aCGH analysis performed to characterise the double segmental imbalances revealed a 14 Mb duplication at chromosome 17q24.2-25.3 and a 769 kb deletion of 7p22.3 (Table 1, Figure 1h). Both affected genomic intervals include a number of genes (Figure 1i)*.*

### Proband 4

This 10-year-old boy, who had presented prenatally with IUGR, was found at clinical evaluation to have dysmorphic facial features including synophrys, a long philtrum, a thin upper lip and ear lobe creases (Figure 1l), along with musculoskeletal anomalies (clynodactyly of the fifth finger and scoliosis); other features were polythelia and cryptorchidism (Table 2 and Additional file 1: Table S1). He also had psychological and cognitive disorders, including hyperactivity, emotional problems and mild intellectual disability. The mother of the proband had similar facial features (synophrys, a long hypoplastic philtrum, a thin upper lip and ear lobe creases), and a monolateral hypoplasic kidney. She has different neurocognitive and psychological disorders, characterised by dyslexia and bulimia.

The aCGH analyses of the proband and his parents revealed an 880 Kb chromosome 19p13.3 duplication in the son and mother who, on the basis of the signals ratio, appeared to be a mosaic (Table 1, Figure 1m).

To estimate the mosaicism rate with a cell-to-cell-based technique, an interphase FISH experiment was carried out on the mother’s nuclei using the CTD-3009K5 BAC clone, which maps within the duplicated region (Figure 1m). Hybridisation signals were scored on 100 nuclei each from mother and son. A signal of either duplicated or increased intensity was identified in all of the son’s cells, whereas in the sample from the mother, cells containing nuclei with this abnormal pattern were prevalent (76%), but the remaining fraction showed a normal hybridisation pattern, confirming the presence of mosaicism for the mutation (Figure 1n).

Forty-three RefSeq genes are located in the duplicated interval (Figure 1o).

## Discussion

CdLS is a genetically heterogeneous disorder, with only 50–60% of clinically diagnosed probands shown to have mutations in one of the known cohesin-associated genes *NIPBL*, *SMC1A*, *SMC3, HDAC8* or *RAD21*. This partial knowledge of the molecular basis of CdLS parallels the wide clinical spectrum, which ranges from extremely mild to severe and includes “borderline” cases, which are often at the interface with other syndromic conditions caused by defects in interconnected cohesion pathways [33]. The genes for cohesin structural subunits and regulators perform crucial roles in the maintenance of genome stability through surveillance of chromatid cohesion throughout the cell cycle, double-strand DNA break repair and long-range regulation of transcription (see [38] for a review). While transcriptional activation and regulation occurs only in cycling and postmitotic cells, the more ancient roles of cohesion in adhesion of sister chromatids and DNA repair are performed throughout the cell cycle (see [39] for a review). Filtering atypical/borderline cases from the overall set of patients with a presumptive or possible CdLS clinical diagnosis is a major challenge. The technology used in this study (aCGH) can identify genomic regions implicated in CdLS and overlapping phenotypes by identifying CNVs that may harbour genes encoding the large number of proteins that may interact with those of the cohesin pathway. Here, we describe four probands with a CdLS-like phenotype and without evidence of mutations in *NIPBL* and *SMC1A*. The aCGH analyses detected unbalanced rearrangements of various sizes and involving chromosomal regions that have not previously been associated with the Cornelia de Lange phenotypic spectrum.

The four imbalances detected were: i) a *de novo* deletion at chromosome 20q11.2-q12 (4.2 Mb); ii) a *de novo* 1p36.23-36.22 deletion (4.8 Mb); iii) a der(7)t(7;17)(p22.3;q24.2) with a 14 Mb duplication in 17q24.2-25.3 and a 769 Kb deletion in 7p22.3; and iv) a familial 880 Kb duplication in 19p13.3, apparently *de novo* in the mother, who presented with a mosaic state and transmitted the duplication to the affected child.

A number of patients carrying pathogenic deletions or duplications partially overlapping those of our cases have been deposited in DECIPHER or ISCA databases. However, the paucity of the accessory clinical data does not permit conclusive comparisons for genotype phenotype correlations.

To the best of our knowledge, only the chromosome 20q imbalance has previously been described in three patients not classified as having CdLS (although they have some features in common with CdLS) who carry a pure 20q deletion that completely or partially overlaps that identified in our proband 1 (Additional file 3: Figure S1 and Additional file 4: Table S3) [40-42]. No patients have been reported in the literature sharing imbalances in the same regions as the other probands. Four patients have been described bearing a terminal or interstitial deletion of 1p36, with proximal breakpoints falling within the region deleted in our proband 2 (patients A, B, and F in [43], and patient D1P3 in [44]), with whom they share a very small part of the deleted region, consistent with the lack of a common clinical picture. Five patients with a duplicated 19p13.3 region have been described, but all carry a deletion of a different genomic region, thus making them not directly comparable to our proband 4 [45-49].

A large number of the genes located in the regions involved in the imbalances identified in this study have been associated with clinical conditions, making it likely that the phenotypes of our probands are the result of contiguous gene syndromes that mimic the multifaceted CdLS syndrome. Our four probands display a high degree of phenotypic heterogeneity, but all share the minimal diagnostic CdLS clinical features including pre- and post-natal growth retardation (Additional file 1: Table S1), mild to severe psychomotor and cognitive impairment and a cranio-facial appearance (Figure 1) characterised by microcephaly or plagiocephaly, long eyelashes, synophrys, thin upper lip and downturned corners of the mouth, long and prominent philtrum, and hirsutism (Table 2).

It is interesting to note that all of the genomic regions involved in the imbalances described here harbour some dosage-altered genes whose functions are directly or indirectly related to those of the known CdLS genes (*NIPBL*, *SMC1*A, *SMC3*) (Figure 1). The genes of interest (*CEP250*, *DSN1*, *MAD2L2*, *APITD1*/*CENP-S*, *RECQL5*, *ANAPC11*, *MED16*, *MBD3*) encode proteins involved in controlling cell cycle progression, including components of the centrosome, the kinetochore, the mitotic spindle assembly checkpoint, the anaphase-promoting complex, and proteins involved in the methylation and unwinding of DNA (Table 3) [50-63].

**Table 3:** Potential functionally relevant genes residing in regions showing imbalances in patients with CdLS-like phenotypes

***Pt***	***Gene***	***Name***	***Function***	***Biological process***	***Ref***	Position*	***Gene alteration***
*1*	*CEP250*	Centrosomal protein 250kDa	Core centrosomal protein required for centriole-centriole cohesion during the interphase of the cell cycle.	Cell cycle progression (centriole-centriole cohesion) ; transcritpion regulation	50	chr20:33506637-33563217	Loss
	*DSN1*	MIND kinetochore complex component, homologue (*S. cerevisiae*)	Kinetochore protein that functions as part of the minichromosome instability-12 centromere complex, required for proper kinetochore assembly and progression through the cell cycle.	Cell cycle progression (kinetochore assembly)	51,52	chr20:34813608-34835644	Loss
*2*	*MAD2L2*	MAD2 mitotic arrest deficient-like 2 (yeast)	Component of the mitotic spindle assembly checkpoint that prevents the onset of anaphase until all chromosomes are properly aligned at the metaphase plate.	Cell cycle progression; DNA repair	53	chr1:11657124-11674265	Loss
	*APITD1/CENPnS*	Apoptosis-inducing, TAF9-like domain 1	Component of multiple complexes, including the Fanconi anemia (FA) core complex, the APITD1/CENPS complex, and the CENPA-CAD (nucleosome distal) complex. Known role in the stable assembly of the outer kinetochore.	Mitotic cell cycle progression; DNA repair; DNA-dependent transcription initiation	54	chr1:10412746-10425459	Loss
*3*	*RECQL5*	RecQ protein-like 5	Member of DNA-helicase with a specific role being coupled to RNAPII transcription and DNA recombination.	DNA helicase activity (DNA repair, transcription regulation)	55,56, 57	chr17:71134545-71174860	Gain
	*ANAPC11*	Anaphase-promoting complex subunit 11	Component of the anaphase.promoting complex/cyclosome (APC/C), a cell cycle-regulated E3 ubiquitin ligase that controls progression through mitosis and the G1 phase of the cell cycle.	Mitotic cellcycle progression	58,59,60	chr17:77442895-77451655	Gain
*4*	*MED16*	Mediator complex subunit 16	Component of the Mediator complex, a coactivator involved in the regulated transcription of nearly all RNA polymerase II-dependent genes	Transcription regulation	61	chr19:867,962-893,218	Gain
	*MBD3*	Methyl-CpG binding domain protein 3	Subunit of the NuRD, a multisubunit complex containing nucleosome remodelling and histone deacetylase activities. It acts as a transcriptional repressor and plays a role in gene silencing	Histone acetylation (transcription regulation, cell cycle progression)	62, 63	chr19:1527678-1543652	Interrupted

*UCSC Genome Browser assembly Mar.2006 (NCBI36/hg18).

In addition to the presence of genes whose products functionally overlap with those of known CdLS genes, further findings supporting the hypothesis that our probands are phenocopies of CdLS include: i) the localisation of the *CEP170* gene which encodes a component of the centrosome [64], within a region (chromosome 1q44) shown to be deleted in a CdLS proband by Borck et al. [11]; ii) the localisation of the *TNKS* gene, involved in sister chromatid cohesion, within the chromosome 8p23.1 region in the CdLS proband reported by Baynam et al. [65].

Moreover, genes with similar functions (*RECQL4*, *BUB1B*, *BUB3*, *CENPA*, *CENPL*, *SMARCA4*, *SMARCC1* and *ATRX*) have also been found to be dysregulated in expression studies of CdLS patients with mutations in *NIPBL*[66].

## Conclusions

We found that a considerable fraction (8%) of the *NIPBL* and *SMC1A* mutation-negative probands with features overlapping with CdLS included in this study were carriers of chromosomal imbalances that may underlie their phenotypes.

The four probands had different chromosomal imbalances, but all involved a number of genes related to progression through the cell cycle and the safeguarding of chromosomal stability (Table 3). Given the similar functions of delangin and the proteins of the cohesin network, we hypothesise that an imbalance of these genes, which probably act in concert with other functionally related genes, contributes to the observed CdLS-like phenotypes.

Our data raise the issue of the complex clinical diagnosis of a syndrome such as CdLS which, through the multifunctional proteins encoded by its known causative genes, has an impact on a myriad of interconnected pathways. It is therefore not surprising that the clinical diagnosis of a CdLS-like phenotype often includes cases of carriers of chromosomal imbalances affecting multiple genomic regions.

## Competing interests

The authors declare they have no competing interests.

## Author’s contributions

CG, LL and PF conceived and designed the study and wrote the manuscript. CP and DR performed the aCGH work and the FISH experiments. CG, JA, MM and SR performed the mutation screening of *NIPBL* and *SMC1A*. CG, CP, JA and PF contributed to interpretation of the aCGH results. AS, GZ, RT, AC and CM recruited the patients and reviewed the clinical data. All of the authors approved the final version of the manuscript.

## Pre-publication history

The pre-publication history for this paper can be accessed here:

http://www.biomedcentral.com/1471-2350/14/41/prepub

## Supplementary Material

Additional file 1: Table S1 Auxological parameters (at birth and at age of evaluation) of probands 1–4 compared with those characteristic of CdLS patients. Click here for file

Additional file 2: Table S2 CdLS diagnostic criteria applied to the described patients. Click here for file

Additional file 3: Figure S1 20q11.2q12 map showing region involved in our patient 1 rearrangement compared to molecularly characterized 20q deletions reported in the literature. Click here for file

Additional file 4: Table S3 Clinical signs of proband 1 compared with those characteristic of CdLS patients and patients carrying an overlapping 20q deletion. Click here for file

## References

[B1] Brachmann W Ein Fall von symmetrischer Monodaktylie durch Ulnadefekt, mit symmetrischer Flughautbildung in den Ellenbeugen, sowie anderen Abnormitäten A case of symmetrical monodactyly representing ulnar deficiency, with symmetrical antecubital webbing and other abnormalities (dwarfish, cervical ribs, hirsutism) Jahrbuch Kinderheilkunde und physische Erziehun 1916 84 225 235

[B2] De Lange C Sur un type nouveau de degenerescence (typus Amstelodamensis) Arch. Med. Enfants 1933 36 713 719

[B3] Van Allen MI Filippi G Siegel-Bartelt J Yong SL McGillivray B Zuker RM Smith CR Magee JF Ritchie S Toi A Clinical variability within Brachmann-de Lange syndrome: a proposed classification system Am J Med Genet 1993 47 947 958 10.1002/ajmg.1320470704 8291538

[B4] Kline AD Krantz ID Sommer A Kliewer M Jackson LG FitzPatrick DR Levin AV Selicorni A Cornelia de Lange syndrome: clinical review, diagnostic and scoring systems, and anticipatory guidance Am J Med Genet A 2007 143A 1287 1296 10.1002/ajmg.a.31757 17508425

[B5] Selicorni A Russo S Gervasini C Castronovo P Milani D Cavalleri F Bentivegna A Masciadri M Domi A Divizia MT Sforzini C Tarantino E Memo L Scarano G Larizza L Clinical score of 62 Italian patients with Cornelia de Lange syndrome and correlations with the presence and type of NIPBL mutation Clin Genet 2007 72 98 108 10.1111/j.1399-0004.2007.00832.x 17661813

[B6] Oliver C Bedeschi MF Blagowidow N Carrico CS Cereda A Fitzpatrick DR Gervasini C Griffith GM Kline AD Marchisio P Moss J Ramos FJ Selicorni A Tunnicliffe P Wierzba J Hennekam RC Cornelia de Lange syndrome: extending the physical and psychological phenotype Am J Med Genet A 2010 152 1127 1135 20425817 10.1002/ajmg.a.33363

[B7] Rohatgi S Clark D Kline AD Jackson LG Pie J Siu V Ramos FJ Krantz ID Deardorff MA Facial diagnosis of mild and variant CdLS: Insights from a dysmorphologist survey Am J Med Genet A 2010 152 1641 1653 20583156 10.1002/ajmg.a.33441 PMC4133091

[B8] Nasmyth K Haering CH Cohesin: its roles and mechanisms Annu Rev Genet 2009 43 525 558 10.1146/annurev-genet-102108-134233 19886810

[B9] Tonkin ET Wang TJ Lisgo S Bamshad MJ Strachan T NIPBL, encoding a homolog of fungal Scc2-type sister chromatid cohesion proteins and fly Nipped-B, is mutated in Cornelia de Lange syndrome Nat Genet 2004 36 636 641 10.1038/ng1363 15146185

[B10] Krantz ID McCallum J DeScipio C Kaur M Gillis LA Yaeger D Jukofsky L Wasserman N Bottani A Morris CA Nowaczyk MJ Toriello H Bamshad MJ Carey JC Rappaport E Kawauchi S Lander AD Calof AL Li HH Devoto M Jackson LG Cornelia de Lange syndrome is caused by mutations in NIPBL, the human homolog of Drosophila melanogaster Nipped-B Nat Genet 2004 36 631 635 10.1038/ng1364 15146186 PMC4902017

[B11] Borck G Redon R Sanlaville D Rio M Prieur M Lyonnet S Vekemans M Carter NP Munnich A Colleaux L Cormier-Daire V NIPBL mutations and genetic heterogeneity in Cornelia de Lange syndrome J Med Genet 2004 41 e128 10.1136/jmg.2004.026666 15591270 PMC1735640

[B12] Gillis LA McCallum J Kaur M DeScipio C Yaeger D Mariani A Kline AD Li HH Devoto M Jackson LG Krantz ID NIPBL mutational analysis in 120 individuals with Cornelia de Lange syndrome and evaluation of genotype-phenotype correlations Am J Hum Genet 2004 75 610 623 10.1086/424698 15318302 PMC1182048

[B13] Miyake N Visser R Kinoshita A Yoshiura K Niikawa N Kondoh T Matsumoto N Harada N Okamoto N Sonoda T Naritomi K Kaname T Chinen Y Tonoki H Kurosawa K Four novel NIPBL mutations in Japanese patients with Cornelia de Lange syndrome Am J Med Genet A 2005 135 103 105 15723327 10.1002/ajmg.a.30637

[B14] Borck G Zarhrate M Cluzeau C Bal E Bonnefont JP Munnich A Cormier-Daire V Colleaux L Father-to-daughter transmission of Cornelia de Lange syndrome caused by a mutation in the 5′ untranslated region of the NIPBL Gene Hum Mutat 2006 27 731 735 10.1002/humu.20380 16799922

[B15] Bhuiyan ZA Klein M Hammond P van Haeringen A Mannens MM Van Berckelaer-Onnes I Hennekam RC Genotype-phenotype correlations of 39 patients with Cornelia De Lange syndrome: the Dutch experience J Med Genet 2006 43 568 575 16236812 10.1136/jmg.2005.038240 PMC2564552

[B16] Yan J Saifi GM Wierzba TH Withers M Bien-Willner GA Limon J Stankiewicz P Lupski JR Wierzba J Mutational and genotype-phenotype correlation analyses in 28 Polish patients with Cornelia de Lange syndrome Am J Med Genet A 2006 140 1531 1541 16770807 10.1002/ajmg.a.31305

[B17] Kline AD Grados M Sponseller P Levy HP Blagowidow N Schoedel C Rampolla J Clemens DK Krantz I Kimball A Pichard C Tuchman D Natural history of aging in Cornelia de Lange syndrome Am J Med Genet C Semin Med Genet 2007 145 248 260 17640042 10.1002/ajmg.c.30137 PMC4902018

[B18] Schoumans J Wincent J Barbaro M Djureinovic T Maguire P Forsberg L Staaf J Thuresson AC Borg A Nordgren A Malm G Anderlid BM Comprehensive mutational analysis of a cohort of Swedish Cornelia de Lange syndrome patients Eur J Hum Genet 2007 15 143 149 10.1038/sj.ejhg.5201737 17106445

[B19] Vrouwe MG Elghalbzouri-Maghrani E Meijers M Schouten P Godthelp BC Bhuiyan ZA Redeker EJ Mannens MM Mullenders LH Pastink A Darroudi F Increased DNA damage sensitivity of Cornelia de Lange syndrome cells: evidence for impaired recombinational repair Hum Mol Genet 2007 16 1478 1487 10.1093/hmg/ddm098 17468178

[B20] Chong K Keating S Hurst S Summers A Berger H Seaward G Martin N Friedberg T Chitayat D Cornelia de Lange syndrome (CdLS): prenatal and autopsy findings Prenat Diagn 2009 5 489 494 19242925 10.1002/pd.2228

[B21] Castronovo P Delahaye-Duriez A Gervasini C Azzollini J Minier F Russo S Masciadri M Selicorni A Verloes A Larizza L Somatic mosaicism in Cornelia de Lange syndrome: a further contributor to the wide clinical expressivity? Clin Genet 2010 78 560 564 10.1111/j.1399-0004.2010.01408.x 20331678

[B22] Hosokawa S Takahashi N Kitajima H Nakayama M Kosaki K Okamoto N Brachmann-de Lange syndrome with congenital diaphragmatic hernia and NIPBL gene mutation Congenit Anom (Kyoto) 2010 50 129 132 10.1111/j.1741-4520.2010.00270.x 20156239

[B23] Oliveira J Dias C Redeker E Costa E Silva J Reis Lima M den Dunnen JT Santos R Development of NIPBL locus-specific database using LOVD: from novel mutations to further genotype-phenotype correlations in Cornelia de Lange Syndrome Hum Mutat 2010 31 1216 1222 10.1002/humu.21352 20824775

[B24] Pié J Gil-Rodríguez MC Ciero M López-Viñas E Ribate MP Arnedo M Deardorff MA Puisac B Legarreta J de Karam JC Rubio E Bueno I Baldellou A Calvo MT Casals N Olivares JL Losada A Hegardt FG Krantz ID Gómez-Puertas P Ramos FJ Mutations and variants in the cohesion factor genes NIPBL, SMC1A, and SMC3 in a cohort of 30 unrelated patients with Cornelia de Lange syndrome Am J Med Genet A 2010 152 924 929 20358602 10.1002/ajmg.a.33348 PMC2923429

[B25] Bhuiyan ZA Stewart H Redeker EJ Mannens MM Hennekam RC Large genomic rearrangements in NIPBL are infrequent in Cornelia de Lange syndrome Eur J Hum Genet 2007 15 505 508 10.1038/sj.ejhg.5201776 17264868

[B26] Ratajska M Wierzba J Pehlivan D Xia Z Brundage EK Cheung SW Stankiewicz P Lupski JR Limon J Cornelia de Lange syndrome case due to genomic rearrangements including NIPBL Eur J Med Genet 2010 53 378 382 10.1016/j.ejmg.2010.08.002 20727427

[B27] Russo S Masciadri M Gervasini C Azzollini J Cereda A Zampino G Haas O Scarano G Tenconi R Di Rocco M Finelli P Selciorni A Larizza L Intragenic and large NIPBL rearrangements revealed by MLPA Eur J Hum Genet 2012 20 734 741 10.1038/ejhg.2012.7 22353942 PMC3376273

[B28] Musio A Selicorni A Focarelli ML Gervasini C Milani D Russo S Vezzoni P Larizza L X-linked Cornelia de Lange syndrome owing to SMC1L1 mutations Nat Genet 2006 38 528 530 10.1038/ng1779 16604071

[B29] Deardorff MA Kaur M Yaeger D Rampuria A Korolev S Pie J Gil-Rodríguez C Arnedo M Loeys B Kline AD Wilson M Lillquist K Siu V Ramos FJ Musio A Jackson LS Dorsett D Krantz ID Mutations in cohesin complex members SMC3 and SMC1A cause a mild variant of cornelia de Lange syndrome with predominant mental retardation Am J Hum Genet 2007 80 485 494 10.1086/511888 17273969 PMC1821101

[B30] Liu J Feldman R Zhang Z Deardorff MA Haverfield EV Kaur M Li JR Clark D Kline AD Waggoner DJ Das S Jackson LG Krantz ID SMC1A expression and mechanism of pathogenicity in probands with X-Linked Cornelia de Lange syndrome Hum Mutat 2009 30 1535 1542 10.1002/humu.21095 19701948 PMC2783874

[B31] Mannini L Liu J Krantz ID Musio A Spectrum and consequences of SMC1A mutations: the unexpected involvement of a core component of cohesin in human disease Hum Mutat 2010 31 5 10 10.1002/humu.21129 19842212 PMC2797832

[B32] Deardorff MA Bando M Nakato R Watrin E Itoh T Minamino M Saitoh K Komata M Katou Y Clark D Cole KE De Baere E Decroos C Di Donato N Ernst S Francey LJ Gyftodimou Y Hirashima K Hullings M Ishikawa Y Jaulin C Kaur M Kiyono T Lombardi PM Magnaghi-Jaulin L Mortier GR Nozaki N Petersen MB Seimiya H Siu VM Suzuki Y Takagaki K Wilde JJ Willems PJ Prigent C Gillessen-Kaesbach G Christianson DW Kaiser FJ Jackson LG Hirota T Krantz ID Shirahige K HDAC8 mutations in Cornelia de Lange syndrome affect the cohesin acetylation cycle Nature 2012 489 313 317 10.1038/nature11316 22885700 PMC3443318

[B33] Deardorff MA Wilde JJ Albrecht M Dickinson E Tennstedt S Braunholz D Mönnich M Yan Y Xu W Gil-Rodríguez MC Clark D Hakonarson H Halbach S Michelis LD Rampuria A Rossier E Spranger S Van Maldergem L Lynch SA Gillessen-Kaesbach G Lüdecke HJ Ramsay RG McKay MJ Krantz ID Xu H Horsfield JA Kaiser FJ RAD21 mutations cause a human cohesinopathy Am J Hum Genet 2012 90 1014 1027 10.1016/j.ajhg.2012.04.019 22633399 PMC3370273

[B34] Hayashi S Ono M Makita Y Imoto I Mizutani S Inazawa J Fortuitous detection of a submicroscopic deletion at 1q25 in a girl with Cornelia-de Lange syndrome carrying t(5;13)(p13.1;q12.1) by array-based comparative genomic hybridization Am J Med Genet A 2007 143 1191 1197 17497725 10.1002/ajmg.a.31737

[B35] Gervasini C Pfundt R Castronovo P Russo S Roversi G Masciadri M Milani D Zampino G Selicorni A Schoenmakers EF Larizza L Search for genomic imbalances in a cohort of 24 Cornelia de Lange patients negative for mutations in the NIPBL and SMC1L1 genes Clin Genet 2008 74 531 538 10.1111/j.1399-0004.2008.01086.x 18798846

[B36] DeScipio C Kaur M Yaeger D Innis JW Spinner NB Jackson LG Krantz ID Chromosome rearrangements in cornelia de Lange syndrome (CdLS): report of a der(3)t(3;12)(p25.3;p13.3) in two half sibs with features of CdLS and review of reported CdLS cases with chromosome rearrangements Am J Med Genet A 2005 137 276 282 16075459 10.1002/ajmg.a.30857 PMC4896149

[B37] Lichter P Cremer T Rooney DE, Czepulkowski BH Chromosome analysis by non-isotopic in situ hybridization Human cytogenetics. A practical approach 1992 Oxford: IRL Press at Oxford University Press 157 192

[B38] Liu J Krantz ID Cornelia de Lange syndrome, cohesin, and beyond Clin Genet 2009 76 303 314 10.1111/j.1399-0004.2009.01271.x 19793304 PMC2853897

[B39] Dorsett D Ström L The ancient and evolving roles of cohesin in gene expression and DNA repair Curr Biol 2012 22 R240 R250 10.1016/j.cub.2012.02.046 22497943 PMC3327610

[B40] Callier P Faivre L Marle N Thauvin-Robinet C Sanlaville D Gosset P Prieur M Labenne M Huet F Mugneret F Major feeding difficulties in the first reported case of interstitial 20q11.22-q12 microdeletion and molecular cytogenetic characterization Am J Med Genet A 2006 140A 1859 1863 10.1002/ajmg.a.31395 16892304

[B41] Iqbal MA Al-Owain M Interstitial del(20)(q11.2q12) - clinical and molecular cytogenetic characterization Am J Med Genet A 2007 143A 1880 1884 10.1002/ajmg.a.31844 17632777

[B42] Hiraki Y Nishimura A Hayashidani M Terada Y Nishimura G Okamoto N Nishina S Tsurusaki Y Doi H Saitsu H Miyake N Matsumoto N A de novo deletion of 20q11.2-q12 in a boy presenting with abnormal hands and feet, retinal dysplasia, and intractable feeding difficulty Am J Med Genet A 2011 155A 409 414 21271663 10.1002/ajmg.a.33818

[B43] Redon R Rio M Gregory SG Cooper RA Fiegler H Sanlaville D Banerjee R Scott C Carr P Langford C Cormier-Daire V Munnich A Carter NP Colleaux L Tiling path resolution mapping of constitutional 1p36 deletions by array-CGH: contiguous gene deletion or “deletion with positional effect” syndrome*?* J Med Genet 2005 42 166 171 10.1136/jmg.2004.023861 15689456 PMC1735995

[B44] D’Angelo CS Gajecka M Kim CA Gentles AJ Glotzbach CD Shaffer LG Koiffmann CP Further delineation of nonhomologous-based recombination and evidence for subtelomeric segmental duplications in 1p36 rearrangements Hum Genet 2009 125 551 563 10.1007/s00439-009-0650-9 19271239

[B45] Byrne JLB Korn GA Dev VG Bunch GM Brooks K Friedman JM Harrod MJE Partial trisomy 19p Am J Hum Genet A 1980 32 64

[B46] Salbert BA Solomon M Spence JE Jackson-Cook C Brown J Bodurtha J Partial trisomy 19p: case report and natural history Clin Genet 1992 41 143 146 1563088 10.1111/j.1399-0004.1992.tb03651.x

[B47] Brown J Horsley SW Jung C Saracoglu K Janssen B Brough M Daschner M Beedgen B Kerkhoffs G Eils R Harris PC Jauch A Kearney L Identification of a subtle t(16;19)(p13.3;p13.3) in an infant with multiple congenital abnormalities using a 12-colour multiplex FISH telomere assay, M-TEL Eur J Hum Genet 2000 8 903 910 10.1038/sj.ejhg.5200545 11175277

[B48] Quigley DI Kaiser-Rogers K Aylsworth AS Rao KW Submicroscopic deletion 9(q34.3) and duplication 19(p13.3): identified by subtelomere specific FISH probes Am J Med Genet A 2004 125 67 72 14755469 10.1002/ajmg.a.20457

[B49] Lybaek H Ørstavik KH Prescott T Hovland R Breilid H Stansberg C Steen VM Houge G An 8.9 Mb 19p13 duplication associated with precocious puberty and a sporadic 3.9 Mb 2q23.3q24.1 deletion containing NR4A2 in mentally retarded members of a family with an intrachromosomal 19p-into-19q between-arm insertion Eur J Hum Genet 2009 17 904 910 10.1038/ejhg.2008.261 19156171 PMC2986486

[B50] Mayor T Hacker U Stierhof YD Nigg EA The mechanism regulating the dissociation of the centrosomal protein C-Nap1 from mitotic spindle poles J Cell Sci 2002 115 Pt 16 3275 3284 12140259 10.1242/jcs.115.16.3275

[B51] Yang Y Wu F Ward T Yan F Wu Q Wang Z McGlothen T Peng W You T Sun M Cui T Hu R Dou Z Zhu J Xie W Rao Z Ding X Yao X Phosphorylation of HsMis13 by Aurora B kinase is essential for assembly of functional kinetochore J Biol Chem 2008 283 26726 26736 10.1074/jbc.M804207200 18640974 PMC2546542

[B52] Cheeseman IM Desai A Molecular architecture of the kinetochore-microtubule interface Nat Rev Mol Cell Biol 2008 9 33 46 10.1038/nrm2310 18097444

[B53] Medendorp K Vreede L van Groningen JJ Hetterschijt L Brugmans L Jansen PA van den Hurk WH de Bruijn DR van Kessel AG The mitotic arrest deficient protein MAD2B interacts with the clathrin light chain A during mitosis PLoS One 2010 5 e15128 10.1371/journal.pone.0015128 21152103 PMC2994903

[B54] Amano M Suzuki A Hori T Backer C Okawa K Cheeseman IM Fukagawa T The CENP-S complex is essential for the stable assembly of outer kinetochore structure J Cell Biol 2009 186 173 182 10.1083/jcb.200903100 19620631 PMC2717651

[B55] Li M Xu X Liu Y The SET2-RPB1 interaction domain of human RECQ5 is important for transcription-associated genome stability Mol Cell Biol 2011 31 2090 2099 10.1128/MCB.01137-10 21402780 PMC3133350

[B56] Islam MN Fox D 3rd Guo R Enomoto T Wang W RecQL5 promotes genome stabilization through two parallel mechanisms–interacting with RNA polymerase II and acting as a helicase Mol Cell Biol 2010 30 2460 2472 10.1128/MCB.01583-09 20231364 PMC2863711

[B57] Schwendener S Raynard S Paliwal S Cheng A Kanagaraj R Shevelev I Stark JM Sung P Janscak P Physical interaction of RECQ5 helicase with RAD51 facilitates its anti-recombinase activity J Biol Chem 2010 285 15739 15745 10.1074/jbc.M110.110478 20348101 PMC2871440

[B58] Wäsch R Robbins JA Cross FR The emerging role of APC/CCdh1 in controlling differentiation, genomic stability and tumor suppression Oncogene 2010 29 1 10 10.1038/onc.2009.325 19826416 PMC3102600

[B59] Nilsson J Yekezare M Minshull J Pines J The APC/C maintains the spindle assembly checkpoint by targeting Cdc20 for destruction Nat Cell Biol 2008 10 1411 1420 10.1038/ncb1799 18997788 PMC2635557

[B60] Jin L Williamson A Banerjee S Philipp I Rape M Mechanism of ubiquitin-chain formation by the human anaphase-promoting complex Cell 2008 133 653 665 10.1016/j.cell.2008.04.012 18485873 PMC2696189

[B61] Wathugala DL Hemsley PA Moffat CS Cremelie P Knight MR Knight H The Mediator subunit SFR6/MED16 controls defence gene expression mediated by salicylic acid and jasmonate responsive pathways New Phytol 2012 Epub ahead of print 10.1111/j.1469-8137.2012.04138.x 22494141

[B62] Noh EJ Lim DS Lee JS A novel role for methyl CpG-binding domain protein 3, a component of the histone deacetylase complex, in regulation of cell cycle progression and cell death Biochem Biophys Res Commun 2009 378 332 337 10.1016/j.bbrc.2008.11.079 19041848

[B63] Saito M Ishikawa F The mCpG-binding domain of human MBD3 does not bind to mCpG but interacts with NuRD/Mi2 components HDAC1 and MTA2 J Biol Chem 2002 277 35434 35439 10.1074/jbc.M203455200 12124384

[B64] Hutchins JR Toyoda Y Hegemann B Poser I Hériché JK Sykora MM Augsburg M Hudecz O Buschhorn BA Bulkescher J Conrad C Comartin D Schleiffer A Sarov M Pozniakovsky A Slabicki MM Schloissnig S Steinmacher I Leuschner M Ssykor A Lawo S Pelletier L Stark H Nasmyth K Ellenberg J Durbin R Buchholz F Mechtler K Hyman AA Peters JM Systematic analysis of human protein complexes identifies chromosome segregation proteins Science 2010 328 593 599 10.1126/science.1181348 20360068 PMC2989461

[B65] Baynam G Goldblatt J Walpole I Deletion of 8p23.1 with features of Cornelia de Lange syndrome and congenital diaphragmatic hernia and a review of deletions of 8p23.1 to 8pter? A further locus for Cornelia de Lange syndrome Am J Med Genet A 2008 46A 1565 1570 18470924 10.1002/ajmg.a.32095

[B66] Liu J Zhang Z Bando M Itoh T Deardorff MA Clark D Kaur M Tandy S Kondoh T Rappaport E Spinner NB Vega H Jackson LG Shirahige K Krantz ID Transcriptional Dysregulation in NIPBL and Cohesin Mutant Human Cells PLoS Biol 2009 7 e1000119 10.1371/journal.pbio.1000119 19468298 PMC2680332

