# Supplementary material for: Genomic imbalances in patients with a clinical presentation in the spectrum of Cornelia de Lange syndrome
Source: BMC Med Genet. 2013 Apr 3;14:41. doi: 10.1186/1471-2350-14-41 (PMC3626829; doi:10.1186/1471-2350-14-41)
Supplement: Additional file 3: Figure S1 — 20q11.2q12 map showing region involved in our patient 1 rearrangement compared to molecularly characterized 20q deletions reported in the literature. [file 1471-2350-14-41-S3.pdf]

**Figure S1**. 20q11.2q12 map showing region involved in the patient 1 rearrangement compared to molecularly characterized 20q deletions reported in the literature.

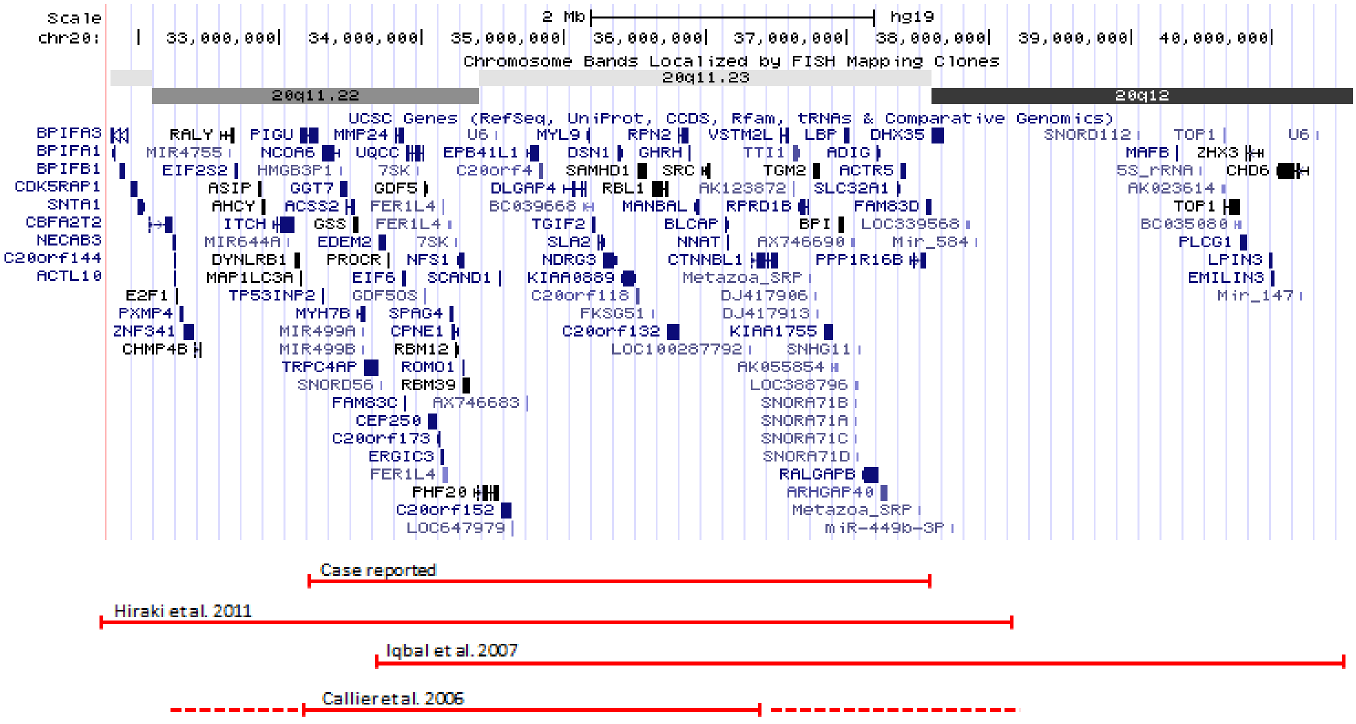


Deleted regions are represented by red bars, dotted lines represent undefined deletions/duplications end regions.
